# The Influence of Environmentally Friendly Flame Retardants on the Thermal Stability of Phase Change Polyurethane Foams

**DOI:** 10.3390/ma13030520

**Published:** 2020-01-22

**Authors:** Dong Liu, Anjie Hu

**Affiliations:** School of Civil Engineering and Architecture, Southwest University of Science and Technology, Mianyang 621010, China; dtld123@126.com

**Keywords:** polyurethane foam, thermal property, phase change material, flame retardant

## Abstract

To improve thermal insulation, microencapsulated phase change materials (micro-PCMs), expandable graphite (EG), and ammonium polyphosphate (APP) were introduced into polyurethane foam (PUF) to enhance the thermal stability and improve the thermal insulation behavior. The morphology of the PUF and micro-PCM was studied using a scanning electronic microscope (SEM), while the thermophysical properties of the PUF were investigated using a hot disk thermal constants analyzer and differential scanning calorimetry (DSC). The thermal stability of the PUF was investigated by thermogravimetric analysis (TGA), and the gas products volatilized from the PUF were analyzed by thermogravimetric analysis coupled with Fourier transform infrared spectrometry (TGA-FTIR). The results revealed that the thermal conductivities of the PUF were reduced because micro-PCM is effective in absorbing energy, showing that the PUF functions not only as a thermal insulation material but also as a heat sink for energy absorption. Moreover, the EG and APP were found to be effective in improving the thermal stabilities of the PUF, and the optimized formulation among EG, APP, and micro-PCMs in the PUF showed a significant synergistic effect.

## 1. Introduction

Widely used for sound absorption [1,2,3], furniture [3,4], and insulating materials [5], polyurethane foam (PUF) plays an important role in our daily life. Compared with other insulating materials, PUF has the advantage of low thermal conductivity, high mechanical and chemical stability at both high and low temperatures, and the ability to form sandwich structures with various facer materials [6,7].

The properties of PUF depend on the density, structure, and geometry of the foam, and a large number of fillers are required to get the desired properties [8,9,10]. To improve the thermal characteristics of PUF, adding phase change material (PCM) into PUF has been studied since the 1990s [11,12,13], since PCM can absorb or release significant latent heat while the temperature of the material stays almost constant in the process of phase change [11,14]. Among the investigated PCMs, the paraffin shows desirable properties, such as high heat of fusion, little or no supercooling, self-nucleating behavior, and thermal and chemical stability. However, liquid paraffin has a relatively low viscosity, and it has to be kept in a closed tank or container to prevent leaching. Microencapsulation of paraffin provides a perfect way to solve the leaching problem. Microcapsules containing the polar phase change material (PCM) n-dodecanol were synthesized by in situ polymerization, using melamine-formaldehyde resin as a shell and styrene-maleic anhydride copolymer (SMA) as an emulsifier. Results show that anionic SMA emulsifier is suitable for the encapsulation of n-dodecanol [15]: when the mass ratio of emulsifier to n-dodecanol is 4.8%, the phase change latent heat and encapsulation efficiency reaches the maximum values of 187.5 J/g and 93.1%, respectively. A series of PU microcapsules containing the PCM of n-octadecane was successfully synthesized, using an interfacial polymerization in SMA dispersion with diethylene triamine (DETA) as a chain extender reacting with toluene-2,4-diisocyanate (TDI), and the average diameter of the microencapsulated phase change materials (micro-PCMs) was in the range of 5–10 μm. Earlier research showed that PU-shells regularly fabricated with the influence of SMA varied with a weight ratio from 1.0% to 4.0% in diameter, encapsulating ratio, release properties, and thermal stability [16].

Another drawback of PUF and paraffin is that, like other organic materials, their flammability is high and the flame-spreading rate is fast, which limits their use due to the high possibility of a fire hazard. Hence, a flame retardant should be added to improve the thermal stability of PUF and paraffin. There are generally two kinds of flame retardants. The first are halogen-containing flame retardants, which are most effective at improving thermal stability. However, they always release a large amount of noxious material during their decomposition, which has brought ecological and physical problems. The second kind are halogen-free flame retardants, such as EG and decabrominated dipheny ethane (DBDPE). The effect of the retardants is influenced by many factors. In the literature [17], the EG/PU composites prepared with their method showed a V-0 flame retardance level, whereas EG/PU composite prepared by conventional blending only showed a V-2 flame retardance level. Results in the literature [9] showed that the flame-retardant efficiency got better with the increase in the density of PU foam at the fixed EG weight percent, or with an increase in the EG weight percent at the fixed foam density. According to the literature [8], when the flame-retardant loadings were 20 wt%, the limiting oxygen index (LOI) value of DBDPE-filled rigid polyurethane foam (RPUF) increased to 33 vol%, while the EG-filled RPUF reached 41%. However, when they were simultaneously added into RPUF, there was not any flame-retardant synergistic effect. In the literature [18], they tested polystyrene composites with 21.4 wt% ammonium polyphosphate, and found that the peak of the heat release rate was reduced by 77.5% as compared to those of virgin polystyrene. In the literature [19], it was found that the peak heat release rate of PEG could be decreased by 19.2% by introducing graphene aerogels (GA) with 1.60 wt% and phosphorylated polyvinyl alcohol (PPVA) with 15.0 wt%. In the literature [20], it was found that, with 0.25 phr of functionalized graphene oxide (fGO) and 10 phr of EG/dimethyl methyl phosphonate (DMMP), the LOI value of RPUF/fGO composites reached 28.1%, and its UL-94 test reached V-0 rating, showing that the fGO could significantly enhance the flame retardant properties of RPUF composites. Carbon nanotubes and zinc aluminum-layered double hydroxide (CNT/ZnAl-LDH) could improve the thermal degradation stability as well as the fire safety of PU foams [21]. These researches indicate that halogen-free flame retardants can effectively improve the stability of the organic materials, and their effects are mostly influenced by the content of the retardants and the synergistic effect of different retardants. Among these retardants, the EG and APP are widely applied due to their good flame-retardant efficiency and economic efficiency. Thus, we applied them in the PUF, and optimized the content of the retardants for the best effect.

In this work, we focused on the effects of EG, APP, and micro-PCMs on the thermal characteristics of PUF. The EG and the APP were added to increase the thermal stability of PUF synergistically, and micro-PCMs were added to improve the temperature-controlling capability of PUF. Besides thermal stability, the EG can also improve the effectiveness of the energy exchange of PCM because of its high thermal conductivity.

## 2. Experimental Section

### 2.1. Materials

Paraffin (melting temperature T_m_ = 58.15 °C, latent heat 163.31 J/g) was used as the phase change material. The melamine (MA), formaldehyde, and dodecyl sodium sulfate were supplied by Sinopharm Chemical Ltd. (Shanghai, China). The polymeric MDI (methane diphenyl diisocyanate, NCO% = 30.2~32.5, viscosity at 25 °C equals to 150~250 mPa∙s) was purchased from Yantai Wanhua Polyurethanes Co., Ltd. (Yantai, China). The polyether polyol (YD 4450, n°OH = 350 ± 10 mgKOH/g, viscosity at 25 °C equals to 220 ± 50 MPa∙s) was supplied by Hebei Yadong Chemical Group Co., Ltd. (Shijiazhuang, China). The ammonium polyphosphate (APP, average particle size 92% < 10 µm) and expandable graphite (EG, average particle size: 320 mesh) were provided by Hefei Keyan Chemical Material Technology Development Co., Ltd (Hefei, China). Other additives are given as follows: dibutyltin dilaurate (DD), triethylenediamine, triethanolamine, silicone oil, and water.

### 2.2. Preparation of Micro-PCMs

Micro-PCMs were fabricated by in situ polymerization, using a melamine–formaldehyde copolymer as the shell and paraffin as the core material. The detailed fabrication process included the steps of prepolymer solution synthesis, PCM emulsion preparation, and mixing of the prepolymer solution and the PCM emulsion for an in situ polymerization. The resultant microcapsules were filtered and washed with distilled water at 70 °C until a pH of 7 was reached. The wet powders were then dried in a vacuum oven at 80 °C for 12 h to remove the water. The final mass fraction of the paraffin was approximately 70%, and the total latent heat of the micro-PCM was 118.9 J/g.

### 2.3. Fabrication of PUF

The PUF formulations are given in Table 1. Making the PUF, materials of catalysts, surfactants, blowing agents, EG, APP, and micro-PCMs, etc. were added to a YD 4450 blend in a proper mold and stirred intensively for 15 s. After that, the calculated quantity of MDI was added and stirred at 2500 rpm for 10 s at a room temperature of 20 °C. Then the foam was placed in an oven at 60 °C for 24 h.

### 2.4. Characterization of Flame Retardant PUF

The morphology and microstructure of the PUF were observed using an electronic scanning microscope (Hitachi X650, Hitachi Limited, Tokyo, Japan). The latent heat and the phase change temperature of the PUF were studied using differential scanning calorimetry (DSC), which was carried out in an argon atmosphere with the DT-50 (Shimadzu, Quioto, Japan) thermal analyzer, with a heating rate of 10 °C/min and a flow rate of 30 mL/min. The thermal conductivities of the PUF were tested by the hot disk thermal constants analyzer. A constant voltage was applied, and a thermocouple recorded the temperature rise by circulating a current along the platinum wire. The precision of the thermal conductivity and the thermal diffusivity were ± 2.0% and ± 5.0%, respectively. The thermal stability properties of the PUFs were tested by a TGA Q5000 IR thermogravimetric analyzer (TA Instruments, New Castle, PA, USA). In the test, the samples were heated from room temperature to 600 °C with a linear heating rate of 20 °C/min in a nitrogen atmosphere. The thermogravimetric analysis-Fourier transform infrared spectrometry (TGA-FTIR) of the sample was performed using the DT-50 instrument interfaced to the Varian 2000 FTIR spectrometer. About 3–5 mg of the product was put in an alumina crucible and heated from 30 to 600 °C. The heating rate was set as 20 °C/min in a nitrogen atmosphere. 

## 3. Results and Discussion

### 3.1. SEM Photos of Micro-PCMs and Pure PUF Structure

**Figure 1:**
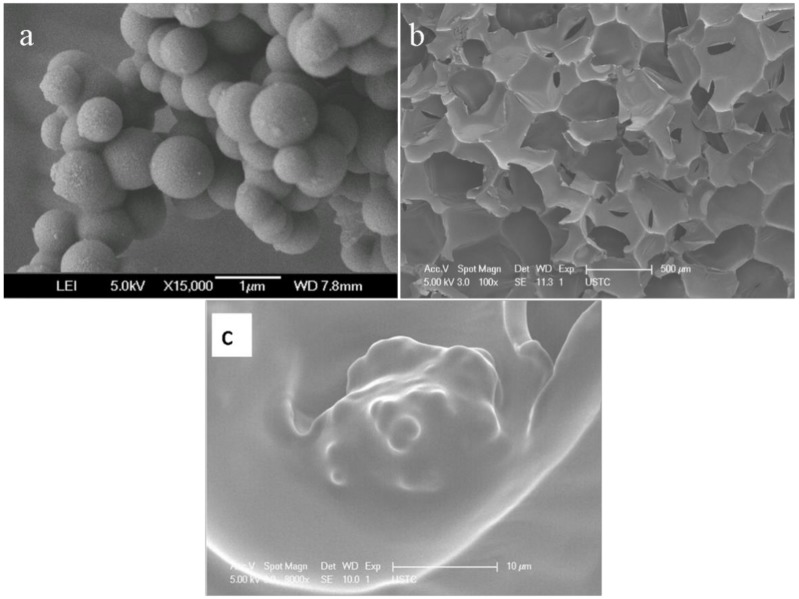
SEM photographs of the micro-PCM (**a**) and PUF (**b**,**c**).

Figure 1a shows the particle profiles of the micro-PCMs. The micro-PCMs have a spherical shape, and the microcapsules range in size from 0.3 to 0.8 µm. The entire honeycomb structure of the pure PUF is shown in Figure 1b, and the honeycomb structure can contain the additives, such as EG, APP, and micro-PCMs. Observing the PUF (Figure 1c), it can be found that the admixture inserted into the pure PUF, showing that the PUF could be used as a supporting material for the admixture.

### 3.2. The Thermal Conductivities of the PUFs

**Figure 2:**
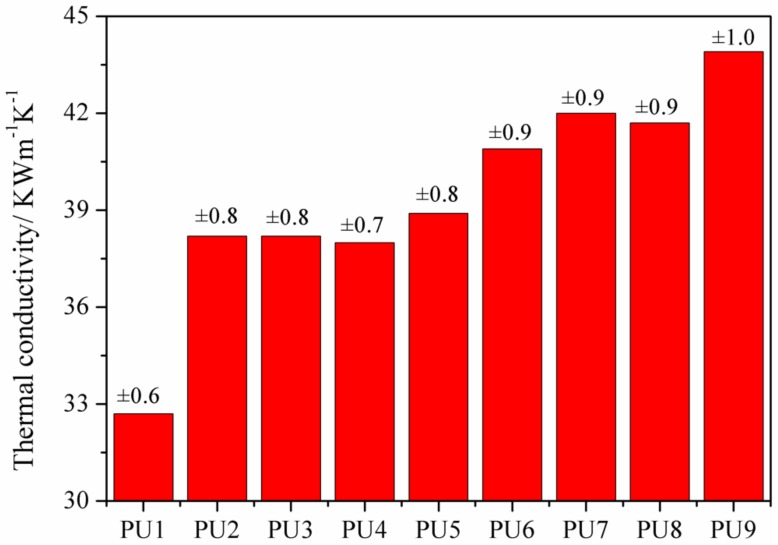
The thermal conductivities of PUFs.

As a widely used thermal insulation material, the heat transfer efficiency of the PUF is the most important property in the application of the material [22,23]. The heat transfer efficiency of PUF is characterized by thermal conductivity [24]. The tested thermal conductivities of PUFs with different formulation are listed in Figure 2, which show that both the additions of EG and APP increase the thermal conductivities of PUFs. However, the increases in the conductivities are relatively small compared to the conductivity of the pure PUF. According to the heat transfer theory, the thermal conductivities of PUFs are determined by the thermal conductivity of the foams and gases within the cell, the radiative heat transfer among these foams, and the convection heat transfer within the cells. Filler particles, including EG and APP, not only fit into the structure of the foam but also lie within the cells, which increases the thermal conductivity of foams and cell gases. However, the filler particles also decrease the radiative and convection heat transfer within the cells, which reduces the effects of the heat transfer enhancement. This conclusion also can explain the result that the thermal conductivities change indistinctively when increasing the content of EG in the PUF. Double side effects also exist in adding the micro-PCMs. When the micro-PCMs are added to the PUF, the thermal conductivities increase, due to their capability for absorbing energy. However, this effect does not reduce the thermal insulation of the PUF, because the energy is restricted into the phase change material.

### 3.3. The Latent Heats of the PUFs

**Figure 3:**
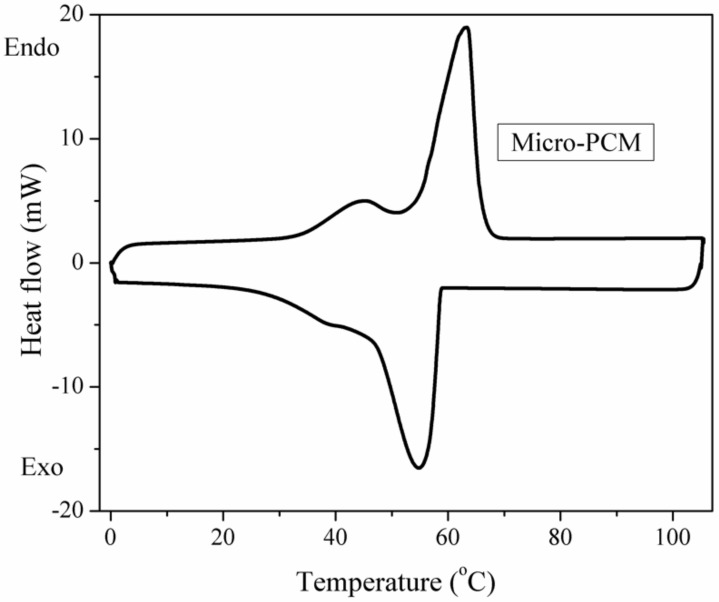
The DSC curves during thermal cycling of micro-PCM.

**Figure 4:**
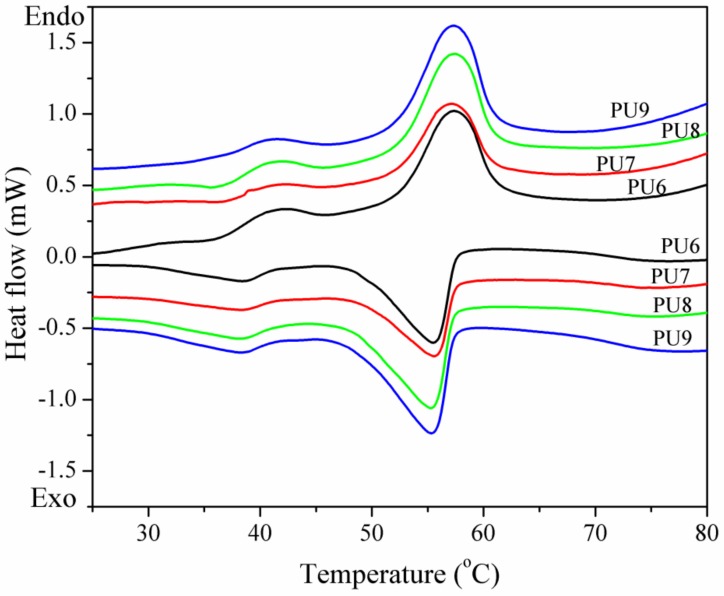
The DSC curves during thermal cycling of PUFs.

The latent heats of the PUFs are tested by the DSC. The DSC curves of the micro-PCM and PU6 to PU9 are exhibited in Figure 3 and Figure 4, respectively. It can be seen that there are two peaks on the DSC curves. The sharp peak represents the solid–liquid phase change of the paraffin, and the minor peak is associated with the liquid–solid phase transition of paraffin. The values of the latent heat obtained with DSC curves are presented in Table 1. As shown in the table, the total latent heats of the PUFs are a little lower than the theoretical values, which were calculated by multiplying the latent heat of the dispersed micro-PCMs with their weight percentage. DSC is also used to investigate the changes of latent heat values under conditions of thermal cycling with the temperature from 80 to 25 °C. From Figure 4 and Table 1, we can find that the energy was released when the temperature decreased, which is useful in order to keep a constant temperature for environment and energy saving. These results demonstrate that the PUFs are not only thermal insulation materials but also storage materials for thermal energy.

### 3.4. The Thermal Stabilities of PUFs

**Figure 5:**
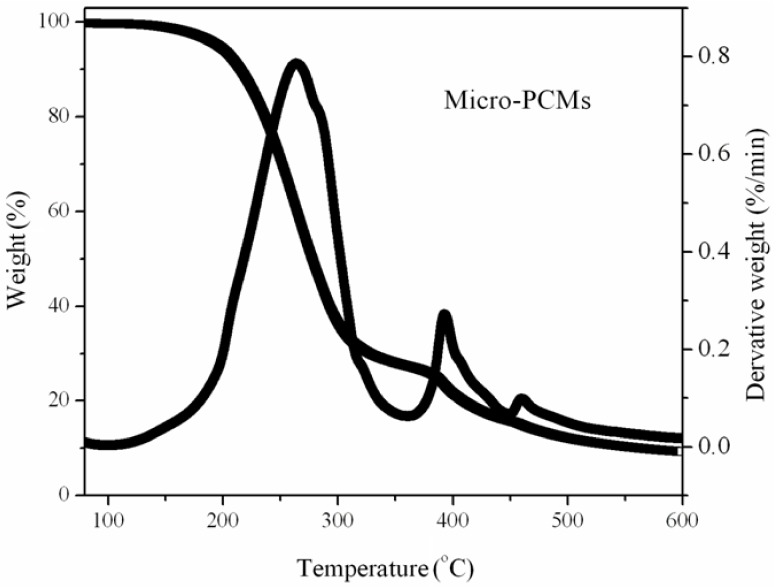
The thermal stability of micro-PCMs.

**Figure 6:**
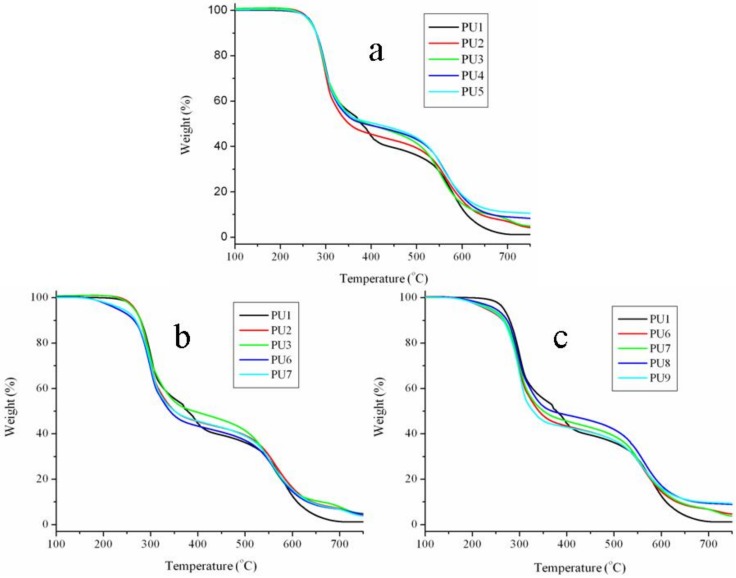
The thermal stabilities of PUFs (**a**) the effects of EG/APP on the thermal stability of PUF, (**b**) the effects of micro-PCMs on the thermal stability of PUF/EG composite, (**c**) the effects of EG/APP on the thermal stability of PUF/micro-PCMs composite.

The thermal stability of the PUF as a thermal insulation material is important for practical applications. Since PUF and micro-PCM both have low thermal stability, they are ignited easily. Improving the thermal stability of PUF is imperative. Figure 5 and Figure 6 show the thermograms of micro-PCMs and PUFs, respectively. The thermal degradation of pure PUF occurs in a two-step process. The first step of degradation is due to the formation of polyol and isocyanate groups. The isocyanates from this deployed condensation reaction are very reactive to dimerize, and they form carbodiimide with the evolution of carbon dioxide. The second step of degradation was due to the further reaction between carbodiimide and polyol to impart the substituted urea [25,26]. From Figure 6a, it can be found that there are no significant changes for the thermal degradation of the PU2 and PU3 when the EG is introduced into the PU1. This is probably due to no reaction/interaction between EG and PUF during the thermal degradation process. However, the rates of thermal degradation for the PU2 and PU3 are accelerated at about 300 to 400 °C in comparison with that of PU1, which may be due to the degradation of intercalated compounds in EG, such as H_2_SO_4_ decomposing to H_2_O and SO_2_ gases and the oxidation of EG, and the distance between the basal planes of EG increasing [27,28]. The structure of EG could prevent the heat and oxygen transfer and improve the thermal stability of the PUF at high temperatures because the EG could cover the surface of the matrix. At higher loadings of EG in PUF, the content of the char layer increases in the temperature range from about 650 to 750 °C, and this char layer could act as a thermal barrier for the PUF and prevent further decomposition. When the mass fraction of APP replaces the EG in equal amounts of PU2 and PU3, the thermal stabilities of the PU4 and PU5 are both increased. This is because the APP can form polyphosphoric acid and carbonaceous residue with PUF at high temperatures, and help in inhibiting the thermal degradation of the PUF. When the micro-PCMs are introduced into the PU2 and PU3, the thermal stabilities of the PU6 and PU7 are both lower than that of PU2 and PU3 (Figure 6b). This is because the thermal stability of the micro-PCMs (listed in Figure 5) is lower than that of PU2 and PU3. However, if part of the EG of PU6 and PU7 is replaced with equal amounts of the mass fraction of APP, the thermal stability of PU8 becomes higher than that of PU6 (Figure 6c), while the PU9 degrades faster than PU7 for temperatures lower than 550 °C and slower for higher temperatures, showing that the ingredients influence the thermal stability improvement due to the synergistic effect of APP and EG. These results show that the APP, melamine and polyol can be effectively taken as an integrated intumescent flame retardant, and the intumescent flame retardant can be thermally decomposed at a higher temperature to form three-dimensional network structures. Besides, the EG acted in great synergistic effect with the APP with a certain formulation in the PUF/micro-PCM system [29].

### 3.5. The Volatilized Products of the PUFs

**Figure 7:**
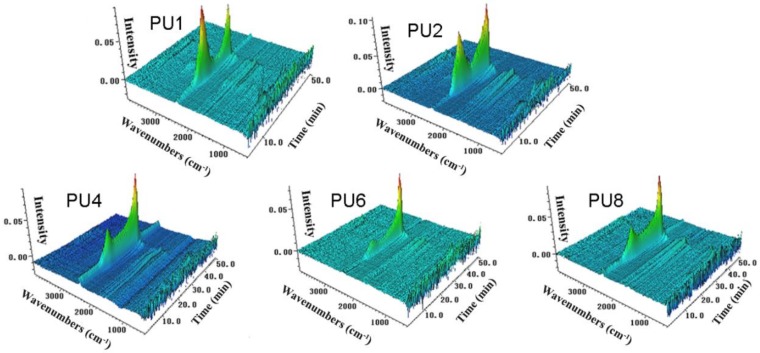
The 3D surface graphs of the FTIR spectra of the evolved gases produced by PUFs.

**Figure 8:**
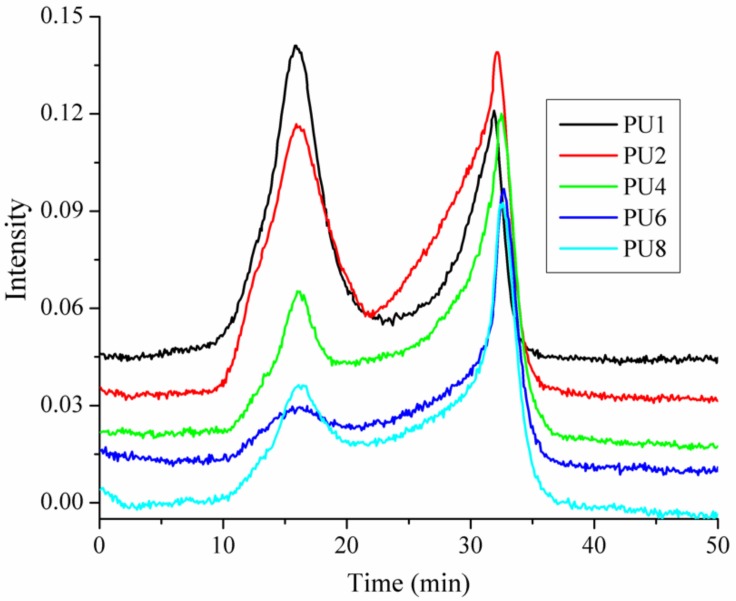
Intensity of characteristic peak versus time for the volatilized carbon dioxide of PUFs.

The TGA-FTIR study is to identify the volatilized products, and the results can provide a better understanding of the thermal degradation mechanisms [30]. The whole range of spectra of the samples is shown in Figure 7. It can be seen that the main decomposition product is carbon dioxide, which is in the range of 2410–2220 cm^−1^. In order to investigate the influence of EG, APP, and micro-PCMs on the thermal degradation of the PUF, the evolution of the intensity of the characteristic peak carbon dioxide is plotted in Figure 8, in which the characteristic peak is at 2356 cm^−1^. It can also be found that the curves of the carbon dioxide show two peaks at about 16 and 32 min, respectively. These peaks are attributed to the depolymerization of PUF to form monomer precursors such as polyol and isocyanates, and the dimerization from isocyanate to carbodiimide and carbon dioxide. The ratio between the second peak value and the first peak value are calculated, i.e., the ratio of PU1 is 0.71, while the ratios of the PU2, PU4, PU6, and PU8 are 1.09, 2.10, 2.84, and 2.23, respectively, showing that EG can highly reduce the carbon dioxide. Comparing the results of PU2 and PU6 with PU4 and PU8, we can also find the reduction of carbon dioxide when replacing EG with APP in PUF. From these results, it can be concluded that EG increased the thermal stability of the PUF, and by replacing part of EG with APP, the efficiency in improving the thermal stability of PU can be further improved.

## 4. Conclusions

The preparation and characterization of PUF containing EG, APP, and micro-PCM are reported. EG and APP were used to improve the thermal stability of PUF, and micro-PCMs were used as the phase change material for thermal energy storage. The SEM images showed that PUF could be an excellent supporting material for EG, APP, and micro-PCM. The thermal conductivity results indicated that the thermal conductivities of the PUF changed owing to the added EG and APP, but the values do not increase continually with the increase of EG and APP contents in the PUF. A possible reason is that the EG and APP could increase the thermal conductivities of the foams and cell gases while decreasing the radiative heat transfer within the foams. The thermal conductivities increased when micro-PCMs were introduced into the PUF because micro-PCMs could absorb the thermal energy; hence, the temperature could be maintained more consistently by the PUF. The DSC results showed that the total latent heats of the PUFs were a little lower than the theoretically calculated values. The TGA and TGA-FTIR results showed that APP and EG have synergistic effects for improving the thermal stabilities of the PUF. When the micro-PCMs are added into PUF, an integrated intumescent flame-retardant system is formed at high temperatures, and optimal formulation among EG, APP, and micro-PCM could provide the optimal synergistic effect in improving the thermal stability of the PUF. All in all, the temperature regulating and controlling effect could be ameliorated owing to micro-PCM, the thermal conductivity of PUF improved with the appropriate amount of EG, and EG and APP showed a great synergistic effect to improve the thermal stability of the PUF/micro-PCM composite.

## Figures and Tables

**Table 1:** Formulations and thermophysical properties of PUFs.

	MDI (g)	YD-4450 (g)	EG (g)	APP (g)	Micro-PCM (g)	Energy Storage Capacity (J/g)	Energy Release Capacity (J/g)
PU1	50	50	0	0	0	0	0
PU2	50	50	10	0	0	0	0
PU3	50	50	15	0	0	0	0
PU4	50	50	8	2	0	0	0
PU5	50	50	13	2	0	0	0
PU6	50	50	10	0	10	7.73	5.87
PU7	50	50	15	0	10	6.58	5.55
PU8	50	50	8	2	10	8.21	7.12
PU9	50	50	13	2	10	6.66	6.02
